# Beeswax as a natural alternative to synthetic waxes for fabrication of PLA/diatomaceous earth composites

**DOI:** 10.1038/s41598-023-28435-0

**Published:** 2023-01-20

**Authors:** Marta Dobrosielska, Renata Dobrucka, Paulina Kozera, Dariusz Brząkalski, Ewa Gabriel, Julia Głowacka, Marek Jałbrzykowski, Krzysztof J. Kurzydłowski, Robert E. Przekop

**Affiliations:** 1 Faculty of Materials Science and Engineering, Warsaw University of Technology, Ul. Wołoska 141, 02-507 Warsaw, Poland; 2 Department of Non-Food Products Quality and Packaging Development, Institute of Quality Science, Poznań University of Economics and Business, Al. Niepodległości 10, 61-875 Poznan, Poland; 3 Faculty of Chemistry, Adam Mickiewicz University in Poznań, 8 Uniwersytetu Poznańskiego, 61-614 Poznan, Poland; 4 Centre for Advanced Technologies, Adam Mickiewicz University in Poznań, Ul. Uniwersytetu Poznańskiego 10, 61-614 Poznan, Poland; 5 Faculty of Mechanical Engineering, Bialystok University of Technology, Ul. Wiejska 45 C, 15-351 Białystok, Poland

**Keywords:** Engineering, Materials science

## Abstract

In this study, injection moulding was applied to produce biocomposites consisting of polylactide (PLA) and amorphous diatomaceous earth used as a filler at different concentrations. Natural wax and synthetic wax were added to improve processing properties, comparing the resulting biocomposites. The use of natural beeswax makes the composite environmentally friendly. The prepared composites contained 2.5, 5, 10 and 15% w/w filler. The test samples have been injection moulded. Rheological, mechanical, surface and other properties were assessed for the fabricated composites. The testing has shown that the use of wax additives has a significant influence on the mechanical properties (tensile strength, flexural strength, impact strength) and the hydrophilicity/hydrophobicity of composite surfaces. The addition of natural wax, especially at lower concentration, has a positive effect on the rheological properties of composites (melt flow rate, MFR), flexural modulus and impact strength. Different composite parameters are modified by different wax types so both natural and synthetic waxes, can be used interchangeably, depending on the required final material characteristics.

## Introduction

Amorphous diatomaceous earth (diatomite, DE) consists mainly of SiO_2_ (weight content of 60–95%) and small quantities of other additives, including ferrous compounds (Fe_2_O_3_) and aluminium compounds (Al_2_O_3_)^1–4^. Diatomite is a great modifier for thermoplastic polymer-based composites, including polyamide or polylactide. Due to its properties (high porosity, low density, high content of amorphous silica), diatomite is used as an adsorbent, germicide, plant protection product and for other applications^5,6^.

Diatomaceous earth is a good alternative to other natural or synthetic fillers. It has a simple chemical composition and a hierarchical micro-/nano-porous structure. Additionally, it can be successfully used as a carrier of active substances. Diatomaceous earth has many advantages, such as porous structure, resistance to acids, neutrality to various types of chemicals and very low density. These characteristics make it possible to find a number of applications, e.g. as an absorbent of harmful gases, as a filtration medium, or a filler for thermoplastics or thermosets to reinforce their structure^7–12^.

Neat diatomite is of natural origin, and hence ideal to be added as a filler for various types of polymers, reducing the cost of manufacturing products, but above all making the production process more sustainable. The use of diatomite to modify polylactide, feedstock of which is also obtained from natural sources, results in an organic biocomposite that is biodegradable under certain conditions. The use of diatomaceous earth as a modifier is known in the literature. Gonzales used diatomaceous earth as a filler material for polylactide, with linseed oil in various concentrations as an additional compatibilizer^13^. On the other hand, Perera used diatomite modified with fluorosilanes and alkylsilanes as fillers for polyurethane to form superhydrophobic surfaces^14^. In contrast, Aguero presented environmentally friendly composites of poly(lactic acid) (PLA) filled with diatomaceous earth (DE), and the composites were prepared by extrusion and injection moulding^15^. A series of compatibilizers/coupling agents, such as 3-glycidyl-oxypropyltrimethoxysilane, epoxy-based styrene-acrylic oligomer, and maleated linseed oil were also used to improve the polymer-filler interaction.

Polylactide is a thermoplastic polymer of natural origin that is susceptible to hydrolytic degradation and industrial composting, making it more environmentally friendly than most petroleum-based polymers^16–19^. Neat polylactide has quite good physical and chemical properties, but the constant need for product improvement has led to additives being used with this polymer to improve its parameters or to obtain other interesting properties; especially, PLA suffers from limited impact resistance and elongation at break. Therefore, improvement of its mechanical properties is constantly sought, which may be achieved through addition of fillers, plasticizers or other types of processing and functional additives. Lee added *N*-ethyl- and *N*-2-hydroxyethyl-substituted maleimide-styrene graft-block copolymer to the polylactide^20^, while in our previous work, it was found that the addition of graphite to PLA is a step towards obtaining material that is cheap and suitable for 3D printing of sliding spare parts^21^. In addition, a polylactide-based material with proper additives can help produce fibre-reinforced composite intended for use in agriculture^22^ or metal alginate-grafted PLA aerogels (PLA-g-M-alginate; M: Ca^2+^, Co^2+^,Ni^2+^, Cu^2+^)^23^. It was previously reported that diatomaceous earth may also be used to reinforce PLA, which is beneficial for both mechanical and thermal properties of such obtained composites^24^. These findings were later confirmed in our studies concerning PLA-based composites^1,7^.

Depending of the additive concentration, the polylactide filled with amorphous diatomaceous earth varies in the melt flow rate, which directly affects the production by injection moulding. To improve the processing properties, various synthetic/natural waxes are used at specified concentrations. The patent application WO2012106006A1^25^ describes the production of a biolaminate made of PLA and natural soy wax. Righetti et al.^26^ described the biocomposite made from polylactide with the addition of plasticizer (tributyl O-acetylcitrate), CaCO_3_, potato pulp and a non-ionic emulsion of beeswax, carnauba wax or modified polypropylene wax. In our previous work^27^, we have determined the effect of the synthetic wax, as well as natural beeswax addition^28^ on the properties of PLA/DE composites. Studies have shown that both types of wax, particularly at high concentrations, improve the processing properties of composites (increase melt flow rate, reduce melt viscosity under shear conditions), and influence many other parameters, including mechanical ones. On top of that, the possible mechanisms of wax interaction with the filler and polymer were discussed, being found highly dependable on the surface treatment of the filler. The effects of wax additives on filler behaviour was studied by Gigante et al. for PLA composites with wheat and rice bran^29^.

**Figure 1:**
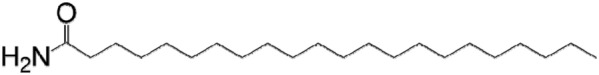
The structural formula of polyamide wax WTH-B.

**Figure 2:**
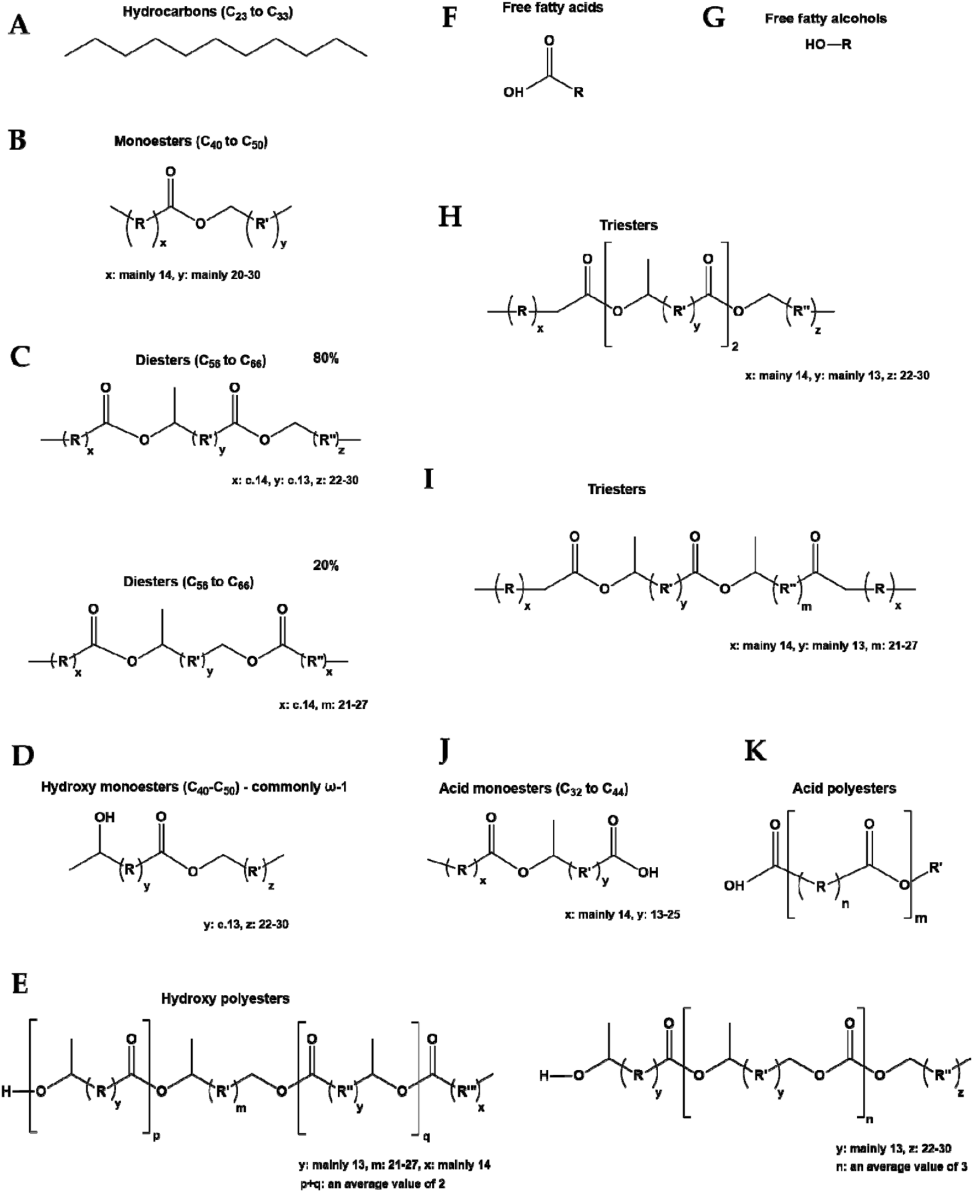
Structural formulae of individual components of beeswax.

Beeswax consists of more than 300 chemical substances, including about 12–16% hydrocarbons with chain length C27–C33, 12–14% free fatty acids with chain length C24–C32, 35–45% fatty acid esters, and other substances. The content of each chemical compound may vary between the different wax-producing bee species and the area in which they occur, due to differences in their diet and genetic predispositions^30^. The structural formula of the polyamide wax WTH-B is presented in Fig. 1, and the composition of beeswax and the structural formulae of the individual components in Table 1 and in Fig. 2.

**Table 1:** Composition of beeswax^31,32^.

Component designation	Name (C_x_ components)	Content [%]
A	Hydrocarbons (C_23_–C_33_)	14
B	Monoesters (C_40_–C_50_)	35
C	Diesters (C_56_–C_66_)	14
D	Hydroxy monoesters (C_40_–C_50_)	4
E	Hydroxy polyesters (no data)	8
F	Free fatty acids (C_22_–C_36_)	12
G	Free fatty alcohols (no data)	1
H-I	Triesters (no data)	3
J	Acid monoesters (C_32_–C_44_)	1
K	Acid polyesters (no data)	2
	Unidentified	6

The PLA/DE composite, to which beeswax is added instead of synthetic wax, not only has better processing properties but also many other parameters. In this work, a number of composites were made with different concentrations of diatomite as a filler material (2.5, 5, 10 and 15%) and different wax content and type. Natural and synthetic waxes were used as modifiers in different concentrations to compare their effect on the properties of the composites produced. Mechanical testing was performed for tensile strength, flexural strength, impact strength and other parameters. In addition, the hydrophobic and hydrophilic character of the surface was examined, the filler material’s dispersion in the polymer matrix was assessed and the waxes’ effect on the diatomite agglomeration was assessed. Thermal stability tests were also performed on the samples obtained, including differential scanning calorimetry (DSC) and dynamic mechanical analysis (DMA). The resulting composites were conditioned at room temperature. The test composites were obtained by widely used injection moulding method.

## Experimental section

### Materials

Polylactide (PLA) of Ingeo 4043D type was purchased from NatureWorks (Minnetonka, MN, USA). Diatomaceous earth (Perma-Guard, Bountiful, UT, USA) was derived from diatomite deposits. Synthetic wax WTH-B Microbeads was sourced from WTH GmbH (Hamburg, Germany). Beeswax was purchased from Spółdzielnia Pszczelarska APIS (Lublin, Poland).

### Preparation of samples

#### Preparation of granulate

The polymer and the filler were homogenized using a laboratory two-roll mill ZAMAK MERCATOR WG 150/280. A portion of 500 g PLA Ingeo™ 4043 D was mixed with diatomaceous earth and wax, until the final concentration of the diatomaceous earth was 5%, 10%, 20% and 30% w/w, 1% and 2% concentration of beeswax or 1% concentration of synthetic wax. The mixing was performed at the rolls temperature of 215 °C for 20 min., to achieve full homogeneity of the concentrates. The masterbatch was granulated with a grinding mill WANNER C17.26 sv and then dried for 24 h at 60 °C.

#### Injection moulding

The prepared masterbatches were diluted 1:1 with PLA directly in the Engel e-victory 170/80 injection moulding machine. Table 2 shows the injection moulding parameters. A holding pressure of linear increment over time was applied. The mould temperature was maintained at 80 °C. Standardized specimens for mechanical tests were obtained according to PN-EN ISO 20753:2019-01. Final system concentrations are shown in Table 3.

**Table 2:** Injection parameters.

	Die	Zone 3	Zone 2	Zone 1	Feed
Temperature [°C]	190.0	195.0	200.0	185.0	40.0

	t [s]	0.0	9.0		
Holding pressure	P [bar]	500.0	1500.0		

Clamping force [kN]	Holding time [s]	Cooling time [s]	Screw diameter [mm]		
800	9.0	50.0	25.0

**Table 3:** Composition of the composites obtained.

	Diatomaceous earth [wt%]	Beeswax [wt%]	Synthetic wax (WTH-B) [wt%]
PLA 4043 D	0	0	0
2.5 DE	2.5	0	0
5DE	5	0	0
10DE	10	0	0
15DE	15	0	0
2.5 DE/0.5 BW	2.5	0.5	0
5DE/0.5BW	5	0.5	0
10DE/0.5BW	10	0.5	0
15DE/0.5BW	15	0.5	0
2.5DE/1BW	2.5	1	0
5DE/1BW	5	1	0
10DE/1BW	10	1	0
15DE/1BW	15	1	0
2.5DE/0.5SW	2.5	0	0.5
5DE/0.5SW	5	0	0.5
10DE/0.5SW	10	0	0.5
15DE/0.5SW	15	0	0.5

### Characterization methods

For flexural and impact strength tests, the obtained materials were injection moulded into dumbbell specimens of type 1B in accordance with PN-EN ISO 527-1:2020-01^33^ and PN-EN ISO 178:2019-06; for tensile tests, 1A specimens were injection moulded^34^. Tensile and flexural tests of the obtained specimens were performed on a universal testing machine INSTRON 5969 with a maximum load force of 50 kN. The traverse speed for the tensile strength measurements was set at 2 mm/min, and for the flexural strength also at 2 mm/min. Charpy impact test (with no notch) was performed on an Instron Ceast 9050 impact-machine according to ISO 179-1^35^. The morphology and microstructure of the prepared composites were observed by scanning electron microscopy (SEM). The imaging was performed in three SE modes. The surface observations were analysed using a Hitachi SU70 scanning electron microscope equipped with an energy dispersive spectrometer (EDS) for chemical analysis. Prior to the observation, the surface samples were coated with electroconductive layer of Au–Pd with a thickness of 3 nm. Observations were conducted in SE mode with an acceleration voltage of 5 kV. Contact angle analyses were performed by the sessile drop technique (5 μL) at room temperature and atmospheric pressure, with a Krüss DSA100 goniometer. The melt flow rate (MFR) was measured using the Instron CEAST MF20 melt flow tester according to the standard EN ISO 1133 at 210 °C and for the load of 2.16 kg. Thermal properties of the materials were examined using a Q1000 Differential Scanning Calorimeter (TA Instruments, New Castle, DE, USA). Samples weighing 8.0 + 0.2 mg were placed in an aluminium hermetic pan. Firstly, the samples were equilibrated at − 90 °C, then heated to 230 °C at a scan rate of 10 °C/min, and cooled to − 90 °C with a scan rate of 10 °C/min. Finally, they were heated again to 230 °C at a scan rate of 10 °C/min. The process was conducted in a nitrogen atmosphere. Using the Universal V4.5A TA software, the glass transition temperature (T_g_) was determined as the midpoint of the glass transition temperature range. The melting temperature (T_m_) was determined as the peak temperatures of cold crystallization and melting, respectively. The rheological properties of the composites were examined using the capillary rheometer Instron CEAST SR10 Smart RHEO 1000. The measurement was carried out at 190 °C using a capillary tube with a diameter of 1 mm and a length of 20 mm. The force was measured using a force converter at specific piston speeds with the range corresponding to apparent shear rates (γ) i.e. 10, 30, 100, 300, 500 and 1000 [1/s].

$$\gamma =\frac{4{R}^{2}}{{r}^{3}}v.$$

*R* bore radius (set in test parameters), *r* die radius (set in test parameters), *v* piston speed calculated from shear rate value. Apparent viscosity was calculated from the formula below:

$$\eta =\frac{\tau }{\gamma },$$

where τ is the apparent shear stress expressed by the following formula:

$$\tau =\frac{Fr}{2\pi {R}^{2}L}.$$

*L* die length (set in test parameters), *F* force measured with load cells.

Light microscopy Images of the surface and fractures of the composites were taken using KEYENCE VHX-7000 digital microscope (KEYENCE INTERNATIONAL, BELGIUM, NV/SA) with VH-Z100R wide-angle zoom lens at × 100 magnification. Images were taken with depth composition and the aid of 3D imaging built-in software. Total coaxial illumination was used. Surface gloss measurements were recorded at 23 °C according to the ISO 2813 standard using a compact gloss meter (GM38, 3Color®, Poland) using 20°, 60° and 85° geometries. The device was calibrated between various specimens measurements using dedicated standard black glass. Three values for each specimen were recorded in gloss units (GU) and averaged. Before the measurement, the samples were stored under the same lighting conditions to prevent possible colour changes. Fourier Transform-Infrared (FT-IR) spectra were recorded on a Nicolet iS50 Fourier trans-form spectrophotometer (Thermo Fisher Scientific) equipped with a diamond ATR unit with a resolution of 0.09 cm^−1^.

Mechanical properties, i.e. ultimate tensile strength, flexural strength, impact strength, and additionally water contact angle were tested for composites, which were previously conditioned at a room temperature (5 days, 23 °C, atmospheric pressure). Imaging of fractures (SEM, EDS, optical microscopy) and thermal tests (DSC, DMTA) were performed on selected composites, i.e. containing different concentrations of the filler to compare the effect of different filling on mechanical and thermal properties.

## Results and discussion

### Analysis of the function groups of modifiers (waxes)

**Figure 3:**
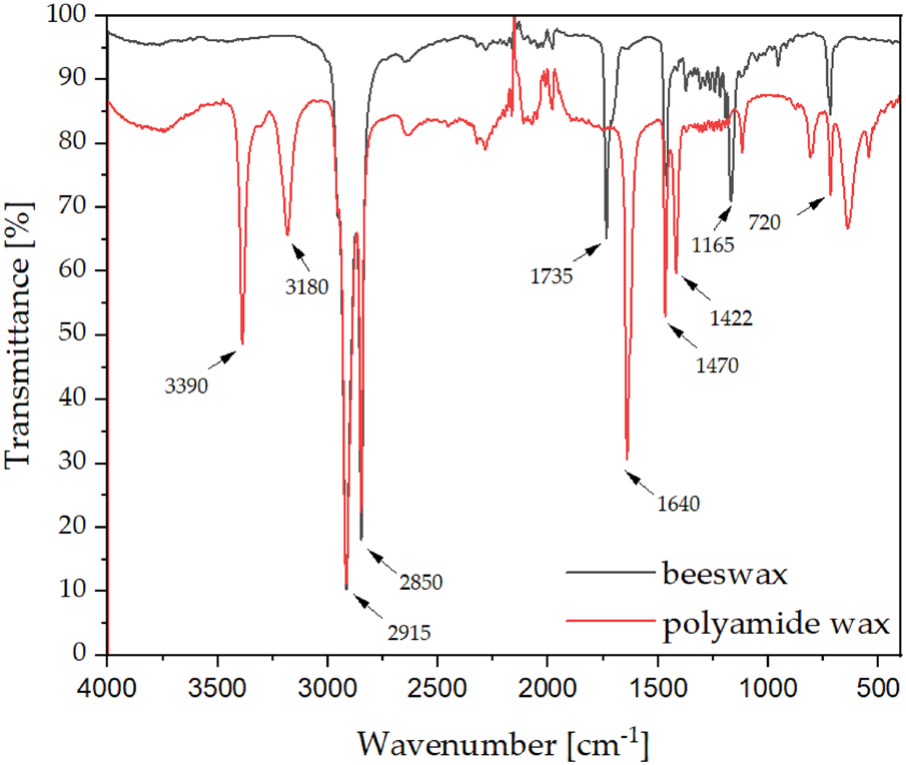
FT-IR spectra of beeswax and polyamide wax.

An analysis of infrared spectroscopy has shown significant differences in structure between the beeswax (natural) and the polyamide wax (synthetic), where beeswax has a simpler structure (Fig. 3). The common bands characterising both types of wax are those at 2915, 2850, 1470, 1422 and 720 cm^−1^, which originate from asymmetric and symmetric stretching of aliphatic hydrocarbons (2915 and 2850 cm^−1^), the in-plane vibrations of aliphatic hydrocarbons (1470, 1422 cm^−1^) and peak due to the rocking of the same vibrational group (720 cm^−1^)^36^. In beeswax, there are also bands at 1735 cm^−1^ and 1165 cm^−1^ due to the presence of carboxyl groups of fatty acids and esters^37,38^. In addition to the common bands with beeswax, synthetic wax is characterised by additional bands at 3390 and 3180 cm^−1^ derived from the amide group CONH_2_ and at 1640 cm^−1^ derived from the carbonyl group.

### Rheology—melt flow rate (MFR) and capillary viscosity

**Figure 4:**
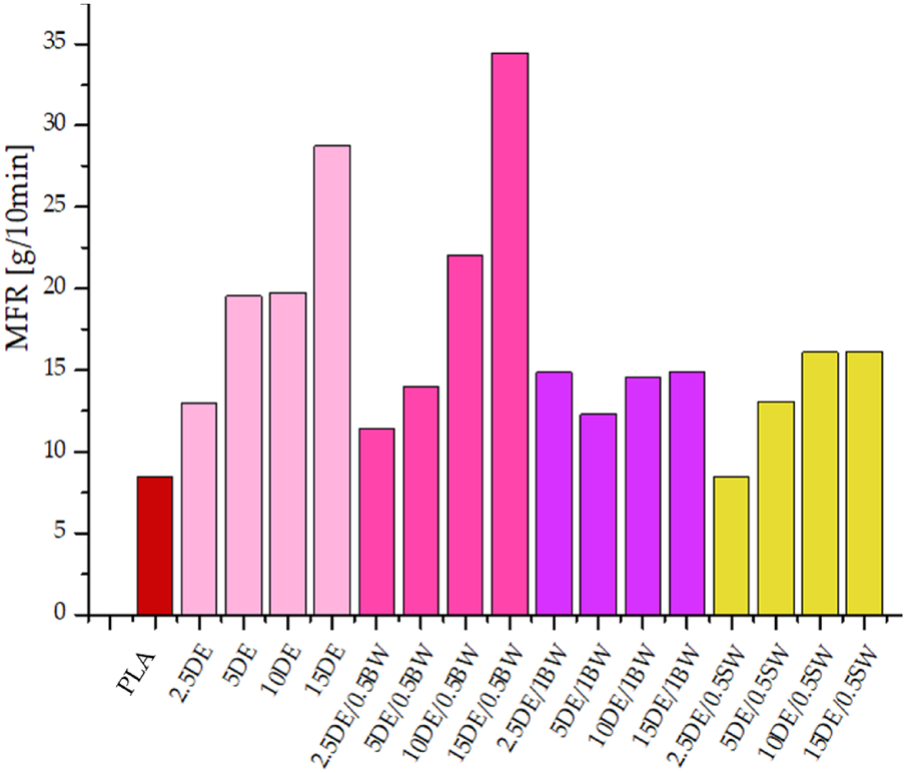
Melt flow rate (MFR) for granulates after injection moulding.

The melt mass-flow rate was measured for ground moulded pieces after the injection moulding. Neat polylactide, according to literature data^39^, has an MFR value of 6 g/10 min at 210 °C. Modification with the original diatomite alone (Fig. 4) added in the range of 2.5–15%, results in a near-consistent increase in melt flow rate to 15 g/10 min, which is equivalent to improved processing performance. Each of the compositions prepared was characterized by increased melt flow rate in comparison to the neat PLA (besides of 2.5DE/0.5SW), with the greatest increase recorded for systems modified with beeswax at a concentration of 0.5%. Such a concentration is optimal, because a further addition (1%) of natural wax results in the drop of MFR, which reaches the value similar to that of samples modified with synthetic wax.

The greatest difficulty in producing high-fill composites is due to high melt viscosity of the processed material. It is important to use additives to facilitate the processing of the material, especially at high concentrations. Beeswax at a concentration of 0.5% per composition has increased the melt flow rate compared to the system containing the same amount of pure diatomaceous earth.

**Figure 5:**
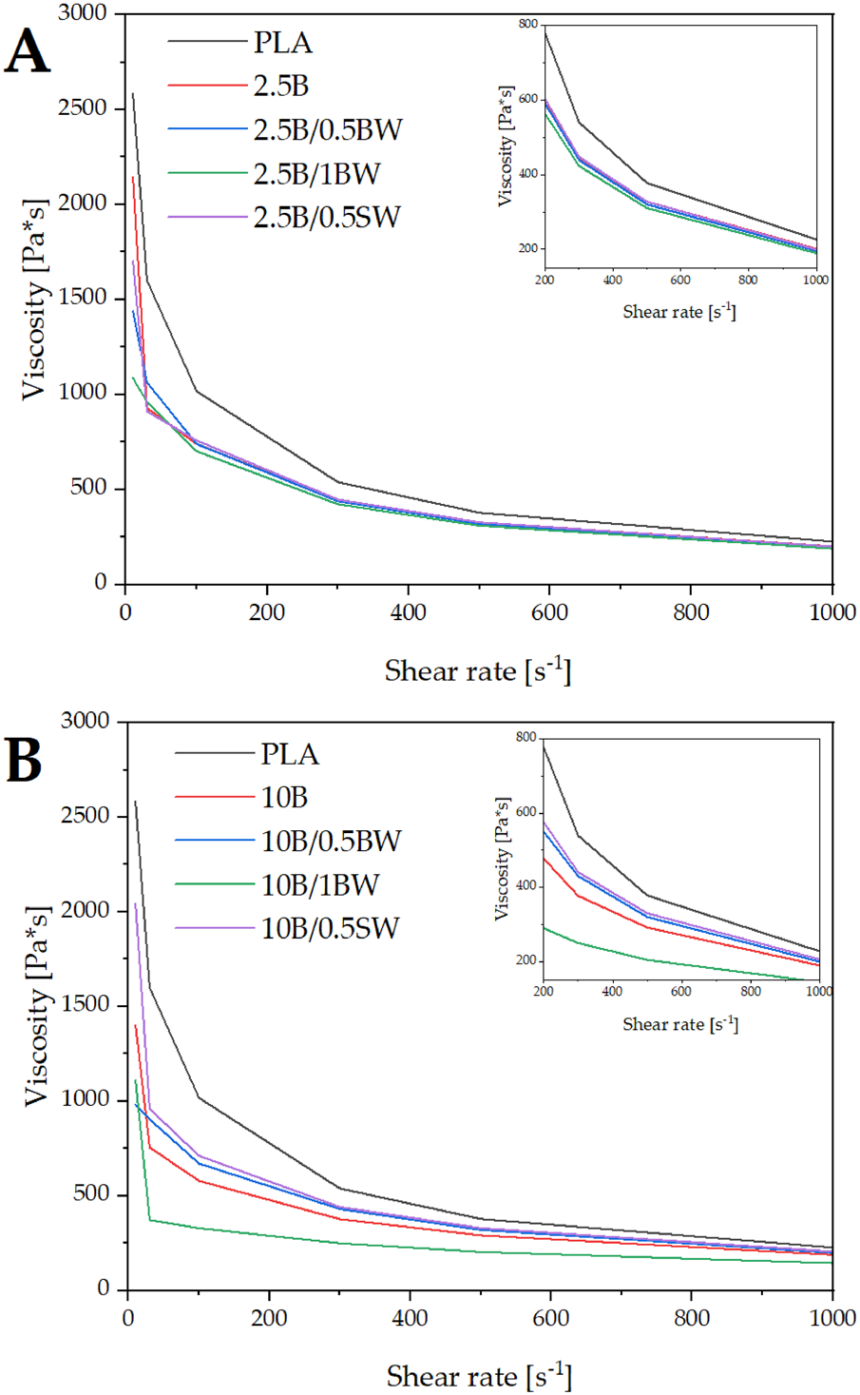
Viscosity of composites after injection moulding; (**A**) composites with 2.5% DE, (**B**) composites with 10% DE.

Figure 5 shows the rheological properties (capillary viscosity) of the compositions with different filler and processing aids content. While the concentration of the filler added increases, the viscosity of the composite is reduced. Within the range of shear rates examined, the molten composite is a non-Newtonian fluid subjected to shear-thinning, which is common for thermoplastics. The viscosity curves of compositions containing 2.5% diatomaceous earth show similar behaviour at increasing shearing rates, i.e. above approximately 200 s^−1^. In this case, the addition of beeswax or synthetic wax at any concentration is irrelevant. At low shearing rates, the viscosity of the samples with 2.5% filler varies considerably and decreases once waxes are added. As beeswax content, the viscosity is further reduced. Interestingly, for 10% filler loading, 0.5% of either wax content, viscosity of the composition is higher than with no wax, while the addition of beeswax at 1% content results in a significant decrease with increasing shear rate. The effect may be due to the particles deagglomeration, which increases filler-matrix contact surface area and smaller particles cause higher viscosity rise in comparison to the agglomerated systems. Under higher shear rates, the matrix-filler interaction becomes less relevant, until viscosity of PLA/DE compositions reaches level almost identical to that of neat PLA.

Diatomaceous earth is a rheological agent for the molten polymer, which means that it increases the melt flow rate of the composite when compared to neat PLA. There is a very sharp decrease in viscosity over the full range of shear rates as the concentration of diatomaceous earth (10%) increases.

**Figure 6:**
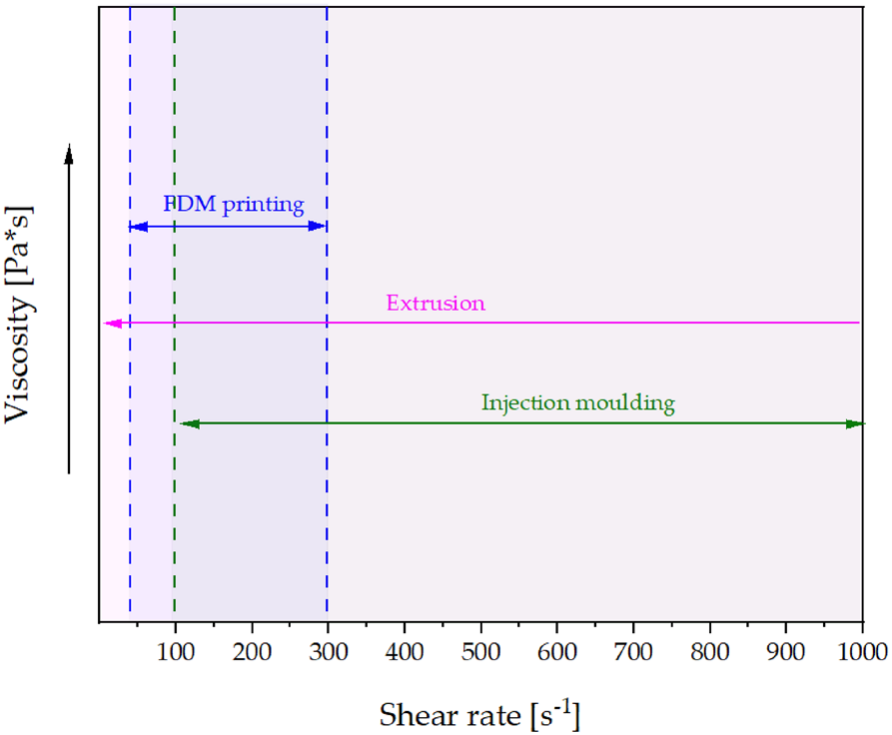
Areas of manufacturing technology: 3D processing area, extrusion technology area, injection moulding technology area.

In direct injection moulding manufacturing which was used in this work to obtain PLA/DE composites, the process shear rates start at 100 [1/s] and often exceed the limit of 1000 [1/s] up to which the capillary rheometer tests were performed as shown in Fig. 6^40^. The difference in viscosity decreases as the shear rate changes. The graphs of all the tested composites flatten out at high shear rates and can be approximated to remain constant as the shear rate increases further during injection moulding. The neat polylactide had the highest viscosity among the analysed samples in the shear rate range tested, which means that by modifying the polymer with diatomaceous earth and waxes, composites with lower processing resistance, or with less stringent processing parameters could be obtained. Figure 6 shows the shear rate ranges accompanying the most common processing, i.e. injection moulding, extrusion and 3D printing^40–42^.

### Mechanical properties

**Figure 7:**
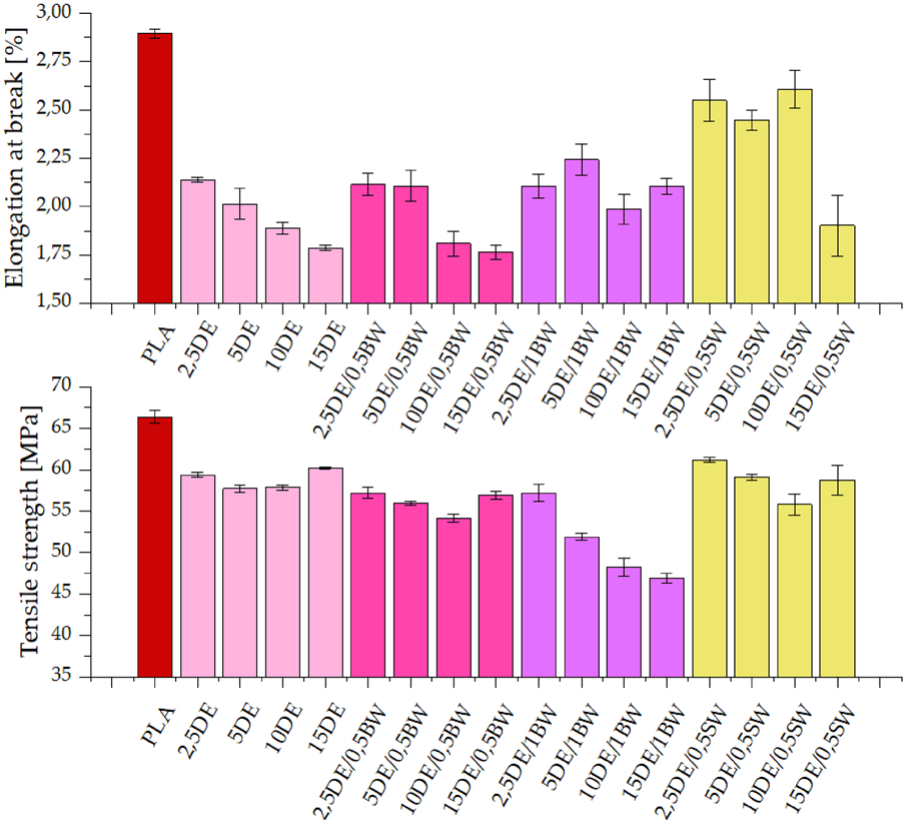
Tensile strength and elongation at break for samples conditioned at room temperature.

The addition of diatomaceous earth to the polylactide results in a decrease in mechanical properties (tensile strength, elongation at break), which is expected due to the particulate filler nature of diatomite, with its relatively high average particle size and low aspect ratio, the filler particles causing discontinuity of the polymer phase and reducing the elasticity of the system. In addition, diatomaceous earth can agglomerate during the composite production, and the deposited particles can cause a concentration of local stresses and consequently a decrease in elongation at break^13^. In the case of modification with diatomaceous earth alone, as well as with addition of DE and 0.5% beeswax, a tendency to decrease the elasticity of the samples with increasing filler concentration is observed (Fig. 7). The situation is different for the other modifications. For the systems containing 1% beeswax, the highest values were recorded for the sample with 5% DE, while for the series without added wax, the values are constant up to a concentration of 10% DE. The decrease in tensile strength for 1% natural wax may be due to the concentration of too much wax at the filler/PLA interphase and a decrease in PLA adhesion to the filler (polymer-filler phase separation).

Tensile strength values for the systems modified with diatomite alone, diatomite and 0.5% beeswax, and diatomite and 0.5% synthetic wax, are at similar levels, both in modifications and in series (Fig. 7). No significant effect of filler concentration on the mechanical strength of the composites was observed, besides of 1BW series, where together with the filler loading, tensile strength dropped due to non-reinforcing behaviour of the filler having no wetting action of the polymer phase, caused by wax overloading, as explained above.

**Figure 8:**
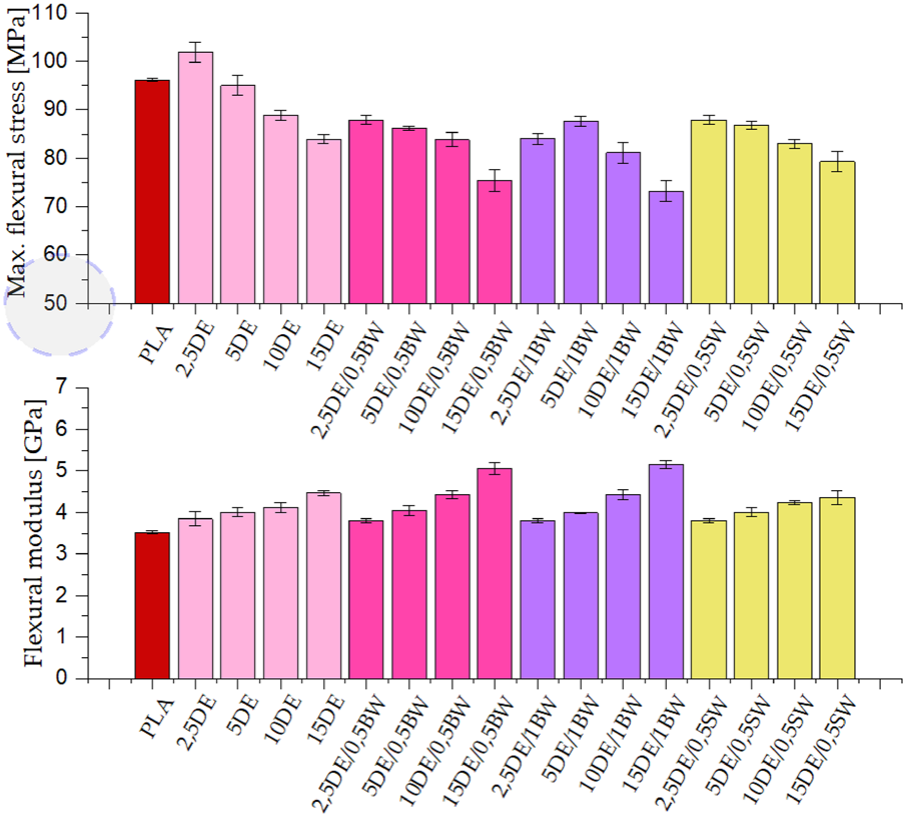
Maximum flexural stress and flexural modulus for samples conditioned at room temperature.

The modification of the polylactide with diatomite has an effect on the flexural strength, which, as already mentioned, is due to the nature of the filler, i.e. the limited adhesion or filler wetting by PLA, lack of flexibility of the filler grains, the filler’s particulate structure and the presence of agglomerates that are structural defects in the composite. Increasing the filler loading to 15% also increases the amount of air injected into the composite due to the porous structure of the diatomite frustules. Therefore, the greater the addition of diatomite to the composite, the lower the flexural stress it is able to carry. This relationship is shown in Fig. 8, where each time the filler loading increases, the maximum flexural stress decreases. A slight positive effect of adding 0.5% beeswax is observed for the sample containing 5% DE, since in this case the maximum flexural stress is the highest and the concentration diatomite in the system is the most favourable. The resulting composites are simultaneously characterized by lower tensile strength, but higher flexural strength. Similar effects have been observed for other PLA-based composite systems before and discussed^43^. This effect may be due to the mixed-mode mechanical stress during the flexural measurements, where both tensile and compressive forces are present, while for tensile strength measurements, the latter are not present. Composites and low compressibility materials, such as concretes, often show higher compressive strength than tensile strength^43,44^.

Even for the lowest diatomite concentrations (2.5%), the flexural moduli are higher than those for the reference sample. With increasing filler concentration, these values increase as well. A similar linear upward trend is observed for systems without wax and with 0.5% beeswax addition. Slightly lower values, with a similar trend, were determined for the other systems. The effect of a decrease in flexural modulus for 1% natural wax compared to 0.5% may also be due to the concentration of compatibiliser at the polymer-filler boundary. While the particles’ rigidity reduces the polymer matrix ability of deforming under load, thus increasing flexural modulus, the large particle size, together with limited matrix-filler wetting action (depending on the methodology of filler pretreatment and composite processing) result in limited capacity of the filler phase to effectively transfer the mechanical load from the matrix phase. Filler pull-out and filler-matrix debonding may occur, which results in mechanical failure of the composite. It can be seen that this effect is more pronounced for higher loadings, where the negative effects of filler agglomeration emerge, as it results in formation of additional mechanical defects in the composite, therefore reducing flexural strength.

**Figure 9:**
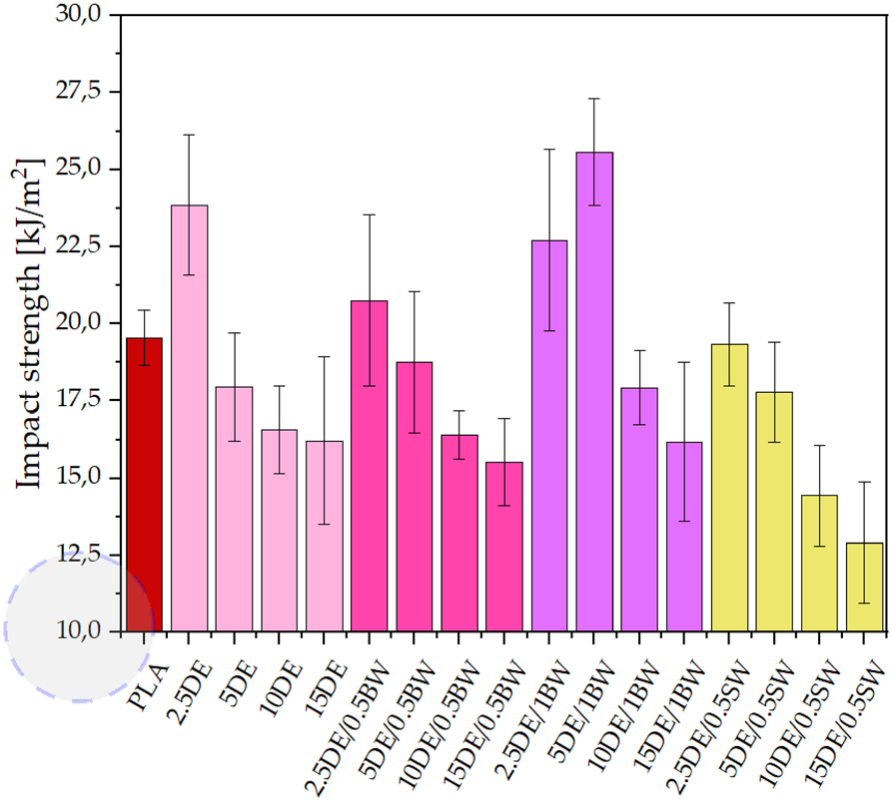
Impact strength for samples conditioned at room temperature.

The impact strength of neat polylactide after injection moulding is around 19 kJ/m^2^. The addition of as little as 2.5% diatomite results in a significant increase in impact strength, up to about 25 kJ/m^2^, demonstrating the positive effect of diatomite alone on the impact strength of the composite. Increasing the diatomite concentration results in an increase in brittleness and hence a decrease in impact strength, which is expected due to the nature of the filler. The addition of 1% beeswax resulted in impact strengths above 25 kJ/m^2^ for 5% DE, which is higher than for pure diatomite and samples with 0.5% beeswax (Fig. 9). The higher error bars obtained for composites containing diatomite, especially at high concentrations, are related to the dispersion of the filler in the polymer matrix and the above mentioned air introduction into the samples, which both are defects of the composite structure.

### Thermal studies

**Figure 10:**
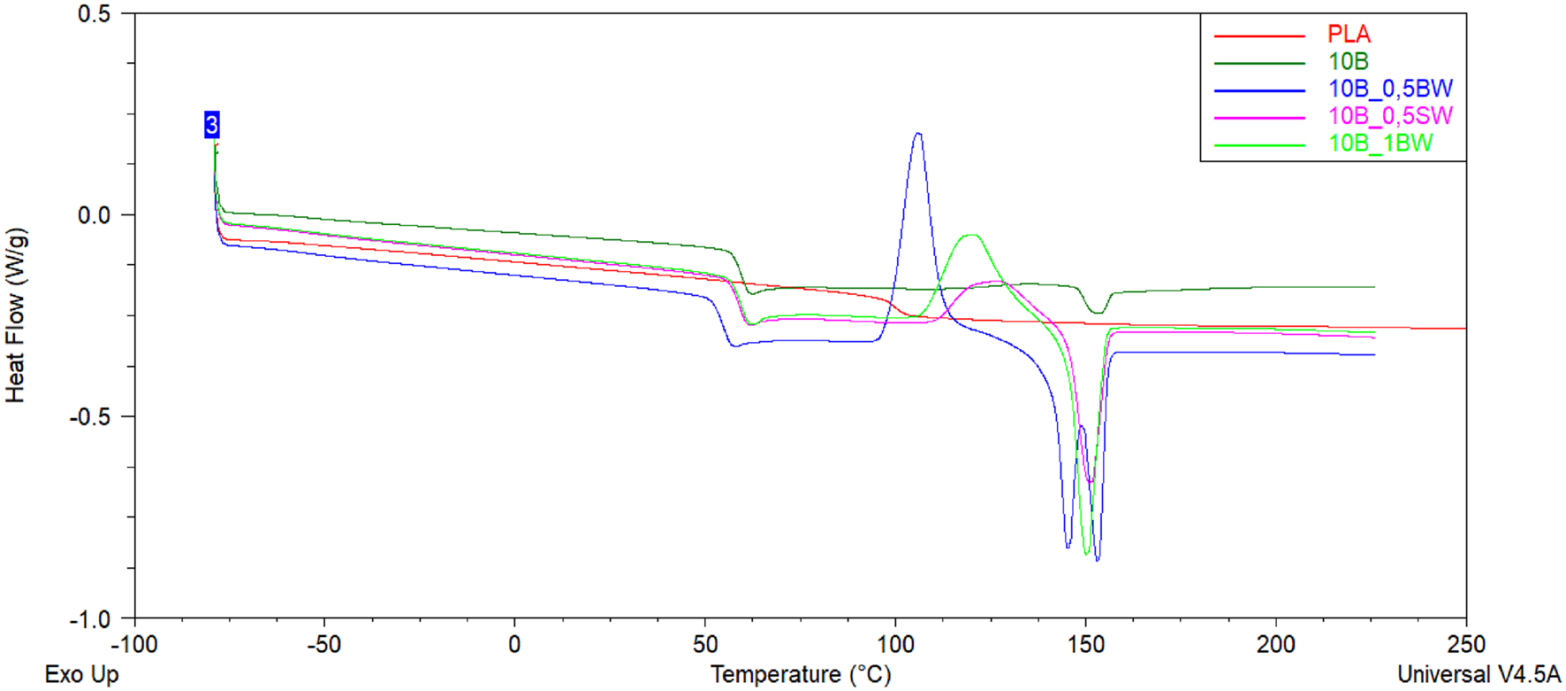
DSC curves of unmodified PLA and 10% DE composites with different type and loading of waxes obtained during second heating cycle.

**Figure 11:**
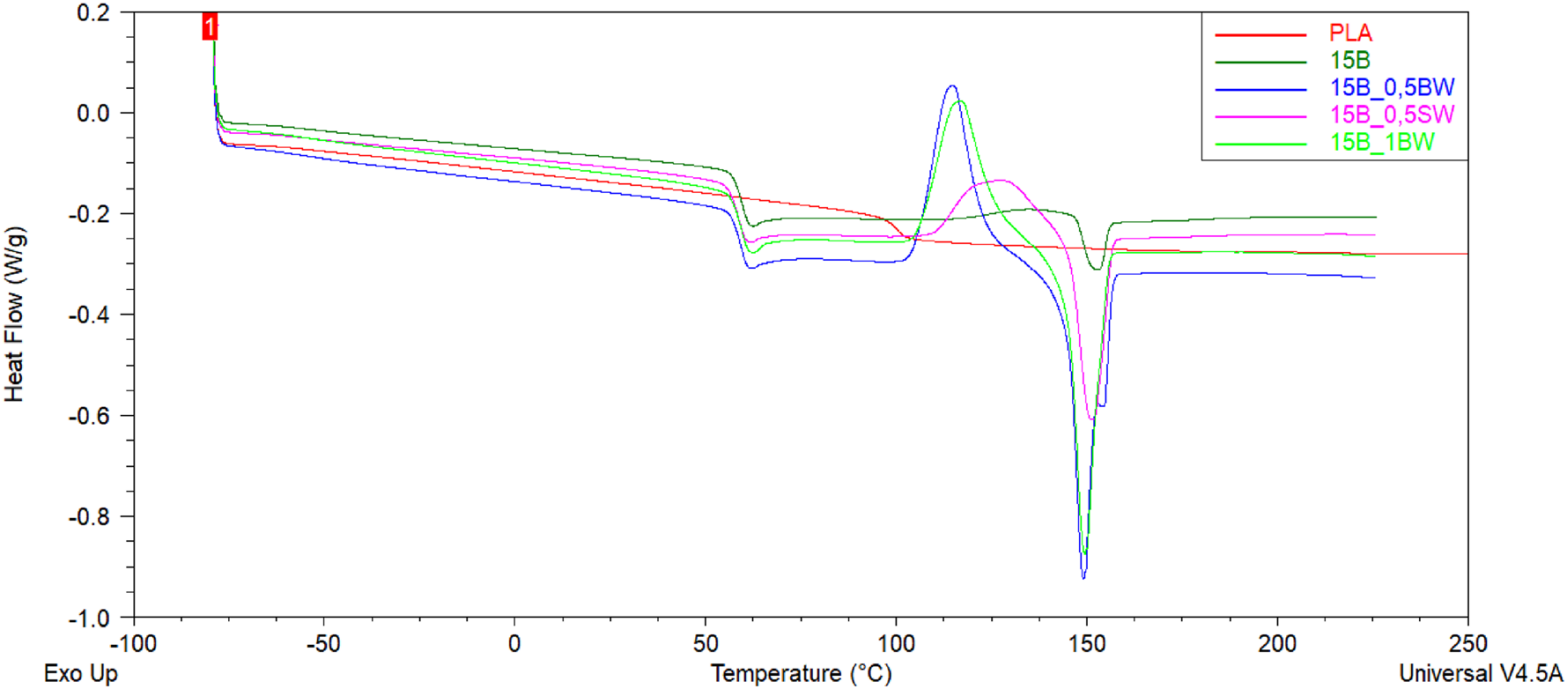
DSC curves of unmodified PLA and 15% DE composites with different type and loading of waxes obtained during second heating cycle.

DSC analysis was performed to investigate the thermal behaviour of PLA and PLA/diatoms composites. Figure 10 and 11 showed the DSC thermograms of PLA and PLA/diatoms composites during the second heating stage. As the temperature increases, each DSC curve exhibits three thermal characteristics: (a) a glass transition temperature (T_g_), (b) a cold crystallization peak (T_cc_), and (c) an endothermic melting peak (T_m_). The values of the glass transition temperature, cold crystallization temperature, specific cold crystallization enthalpy, melting temperature, and specific melting enthalpy are listed in Table 4.

**Table 4:** Thermal characteristics of PLA and composites based on PLA with different weight fraction of diatoms and different kind of waxes.

Sample	2nd heating				
	T_g_ (°C)	T_cc_ (°C)	ΔH_cc_ (J/g)	T_m_ (°C)	ΔH_m_ (J/g)
PLA	N/A	–	–	–	–
10B	59.5	134.5	1.0	153.0	2.3
10B_0.5BW	55.0	105.9	31.3	145.3 and 153.0	31.2
10B_0.5SW	59.0	126.3	11.2	151.0	13.7
10B_1BW	59.6	120.6	20.6	150.1	21.0
15B	59.9	134.0	1.7	152.1	3.5
15B_0.5BW	59.4	114.7	25.1	149.1 and 154.8	25.3
15B_0.5SW	58.8	127.5	14.3	151.2	15.3
15B_1BW	59.5	116.9	23.6	149.6	23.8

The neat PLA used in this study basically does not have a well-defined crystalline phase presence. No T_g_ or T_cc_ was observed, and only a small endothermic event at ~ 100 °C, suggesting either melting of a metastable phase (T_m_) without further crystal perfection and remelting or relaxation of some polymer chains, but without subsequent cold crystallization, as the temperature of this transition is in the typical range of T_cc_. It can be observed that the application of diatoms and waxes changed the character of the DSC curves. For neat PLA, no T_cc_ and T_m_ events were observed. The glass transition temperature for the composites was determined about 60 °C. For the rest of the composites a glass transition temperature, a temperature and enthalpy of cold crystallization peak, and temperature and enthalpy of an endothermic melting peak were determined. It was also found that 10B_0.5BW and 15B_0.5BW composites had two distinct melting peaks (T_m1_ and T_m2_), which can be attributed to the melting of metastable and perfect crystals, respectively^45^. The addition of 0.5% of beeswax reduces the melt viscosity and facilitates wetting action of PLA on the diatomite filler, which results in improved nucleation, which is visible by the occurrence of a well-defined cold crystallization (T_cc_) event. At higher loading of BW or when SW was applied, the nucleation was obstructed and observed at higher temperatures, as most likely too much wax was aggregated on the matrix-filler interphase. Moreover, application of 1%BW or filler loading of 15% affected both the T_cc_ and T_m_, showing that when the cold crystallization occurs at the higher temperature, it prevents formation of the low melting point metastable crystals, and instead, a single melting event is observed, however of slightly lower temperature than that of the perfect crystals for double melting event observed earlier.

### Surface properties

#### Contact angle of the composites’ surface

The contact angle has been measured for systems conditioned at room temperature (Table 5). The reference polylactide is characterized by a hydrophilic surface. Samples containing neat diatomite are characterised by a contact angle in the range of approximately 86°–104° regardless of concentration and conditioning regime, making the surface hydrophobic in most cases. This is due to the additional micro-roughness created by the filler. Composites containing beeswax, have the highest values for systems containing 5 and 10% diatomite. Modification with synthetic wax did not result in significant differences between systems with beeswax and synthetic wax. As the filler concentration increases, the contact angle also increases, reaching more than 95° for the highest concentration of DE.

**Table 5:** Contact angle of the composites after conditioning at room temperature (RT).

Contact angle RT [°]							
PLA				83.2			
2.5DE	102.2	2.5DE/0.5BW	73.8	2.5DE/1BW	89.3	2.5DE/0.5SW	93.6
5DE	96.0	5DE/0.5BW	96.1	5DE/1BW	95.1	5DE/0.5SW	86.3
10DE	99.1	10DE/0.5BW	94.2	10DE/1BW	96.3	10DE/0.5SW	92.9
15DE	85.1	15DE/0.5BW	91.5	15DE/1BW	91.5	15DE/0.5SW	85.8

**Figure 12:**
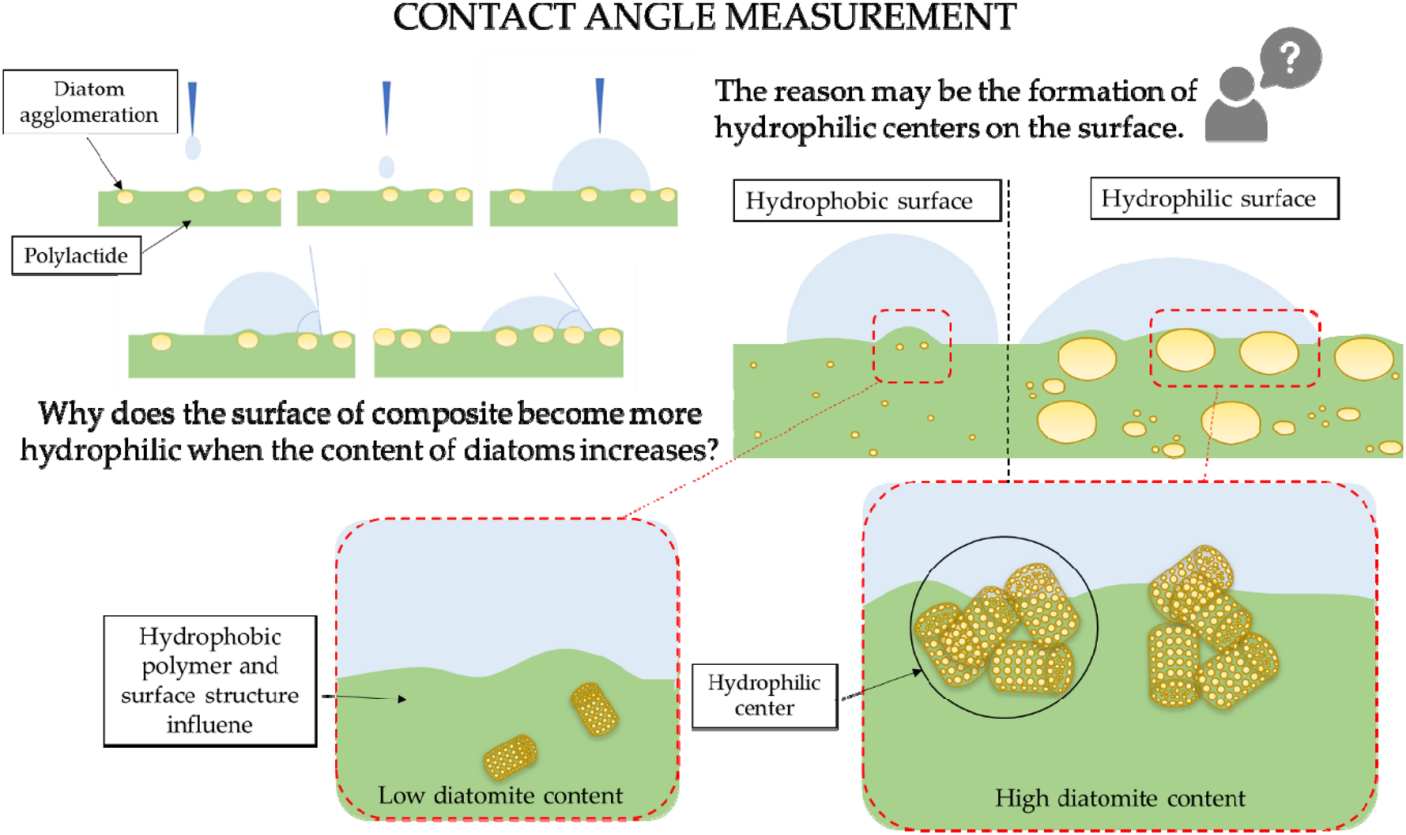
Loading-dependent hydrophilic-hydrophobic nature of PLA/diatomite composites’ surface.

The hydrophobicity of the composites is mainly influenced by the diatomite itself and not by the addition of waxes. Even small addition (2.5%) of diatomite increases the contact angle from 83.2° for the reference polylactide to 102.2°. For such a DE concentration, the increase in hydrophobicity is related to the geometry of the composite surface, i.e. an effect of micro roughness on the hydrophobic–hydrophilic character of the surface is observed. At higher concentrations of diatomite, the decrease in hydrophobicity is related to the formation of hydrophilic centres at the composite/water interface. The hydrophilic centres are formed due to the large accumulation of diatomite, which tends to agglomerate. Diagrams of hydrophilic centres are presented in Fig. 12.

#### Gloss of composite surfaces

The gloss of composite surfaces was analysed for samples conditioned at room temperature. The test was performed at 23 °C in the geometry of 60° since the results were in the range 10–70 GU in accordance with ISO 2813. Systems with 2.5 and 10% filler were selected for comparative testing. All composites are characterized by medium gloss properties. Of all the samples analysed, the low-fill systems have the highest gloss (2.5B–37.9 GU, Table 6), while the addition of a larger amount of diatomite to the composites resulted in a reduction of gloss by more than 40% (10B–21.7 GU), with neat polylactide characterized by the gloss value of 52.1 ± 0.90 GU, which shows the strong influence of neat diatomite on the gloss of polylactide. Key factors affecting the optical properties of diatomite-filled composites are the structural features of the diatomite particles (surface roughness, number of porous silica layers, thickness, and pore diameters) and the refractive index of silica content. The periodic distribution of holes in the diatoms forms a kind of mesh. The periodicity and porous structure allow interaction between light and frustules (photon–matter interaction), so the incident light waves can undergo diffraction, scattering and guiding, and other phenomena. Therefore, the size, number and distribution of the diatom particles in the polymer structure (PLA) influence the change in the gloss of the samples^46^. Referring to structural factors, it can be said that with increasing diatom concentration, the light waves are more scattered, are partially attenuated, so that the detector of the gloss measurement system receives a weaker signal compared to the reflection from the neat polymer, which is interpreted as a lower gloss of the test sample (lower GU value).

**Table 6:** The gloss of composites conditioned at room temperature.

	Gloss [GU]	SD [GU]	Gloss [GU]	SD [GU]
	2.5B		10B	
–	37.87	1.96	21.73	0.12
0.5WP	23.00	0.92	16.20	0.61
1WP	35.36	0.37	24.70	0.78
0.5WS	31.83	0.90	23.60	2.69

This effect is expected due to the nature of the filler and its ability to increase the brittleness and porosity of the composite surface. After the addition of 0.5% beeswax, composites with a much lower surface gloss than without the additive were obtained, i.e. for the 2.5% filler the gloss decreased to 23 GU, while for the 10% filler to 16.2 GU, which is the lowest result obtained. In contrast, a higher addition of the modifier (1%) in the form of natural wax did not result in a loss of surface gloss, as the results before and after modification are within the standard deviation. In the case of a 10% filling, it was even possible to achieve an improvement in gloss compared to the system without modification. Similar but slightly lower results were recorded for composites, to which synthetic wax was added. This leads to the conclusion that, in view of the abovementioned correlations, the addition of beeswax at a higher concentration is advantageous for the surface behaviour of PLA/DE composites.

### Morphology of composite fractures

**Figure 13:**
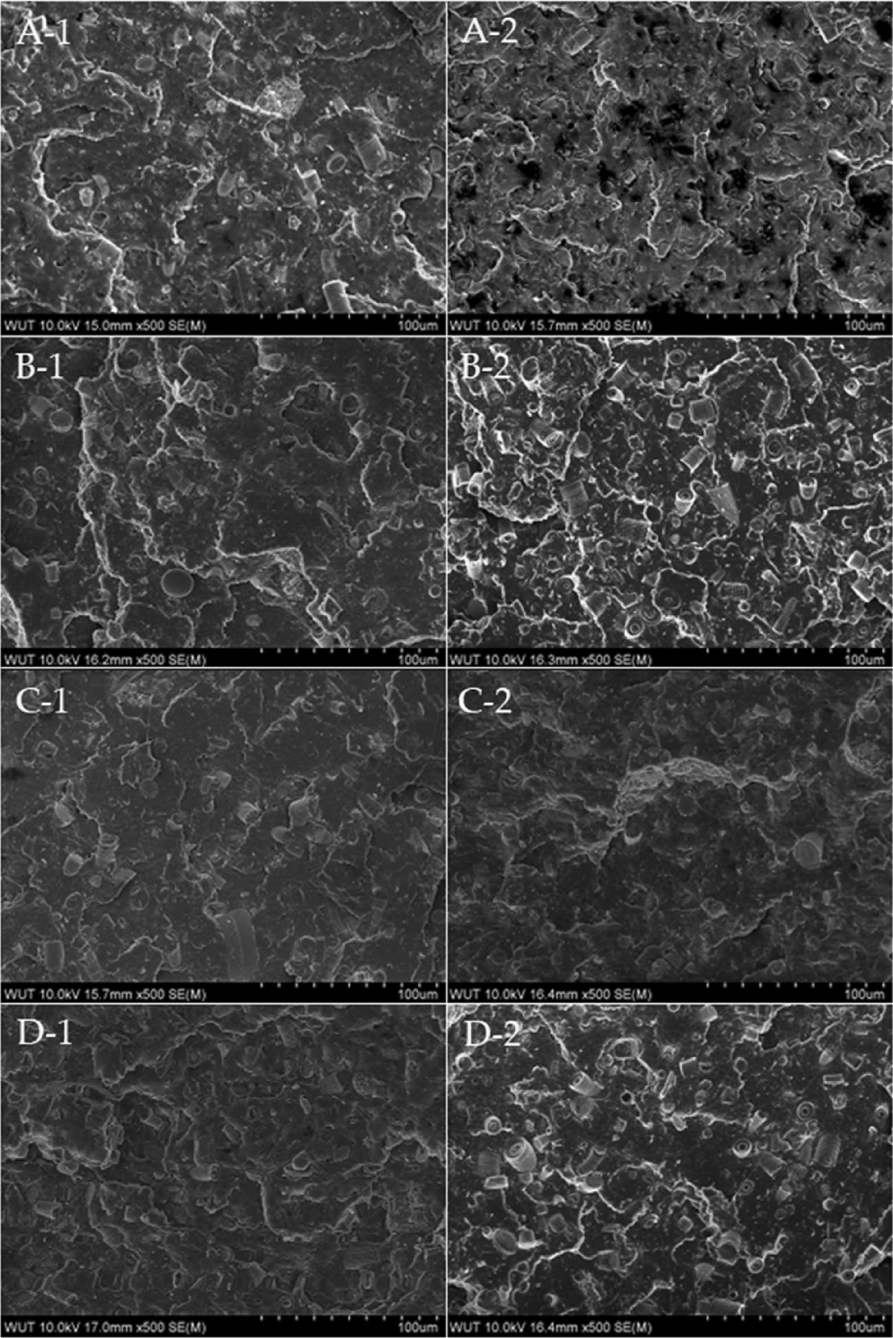
SEM images of composites; (**A-1**) 10B, (**A-2**) 15B, (**B-1**) 10B/0.5BW, (**B-2**) 15B/0.5BW, (**C-1**) 10B/1BW, (**C-2**) 15B/1BW, (**D-1**) 10B/0.5SW, (**D-2**) 15B/0.5SW.

Images taken with a scanning electron microscope have revealed the effects of applied waxes at different concentrations on the filler dispersion in the polymer matrix. The 10% and 15% systems (high-fill) were selected for tests, and the filler dispersion in their matrix was assessed as accurately as possible (Fig. 13). Neat diatomaceous earth embedded in polylactide is characterised by an even distribution of diatom particles for the 10B system. Higher concentration of the filler resulted in more non-uniform edges after the impact test, indicating increased brittleness of the composite. The addition of 0.5% beeswax caused an agglomeration of the diatomite particles, especially for its highest concentration, but this did not negatively affect the mechanical properties of the composite. In this system, the diatoms are present on the surface of the fracture, and not below the crack line as in a system without added wax. In addition, diatom frustules did not break, since whole diatoms can be observed with unchanged shape, which indicates that the addition of wax causes filler pull-out during fracture. The larger, i.e. 1% addition of beeswax had a different effect on the fracture structure of the composite. The agglomeration still occurs, even at lower concentrations, which shows that the higher the concentration of beeswax, the stronger the tendency towards agglomeration, which is not present only at the highest filling. The addition of synthetic wax had a negative effect on the dispersion of the filler in the matrix even at lower concentrations although 0.5% wax was added.

**Figure 14:**
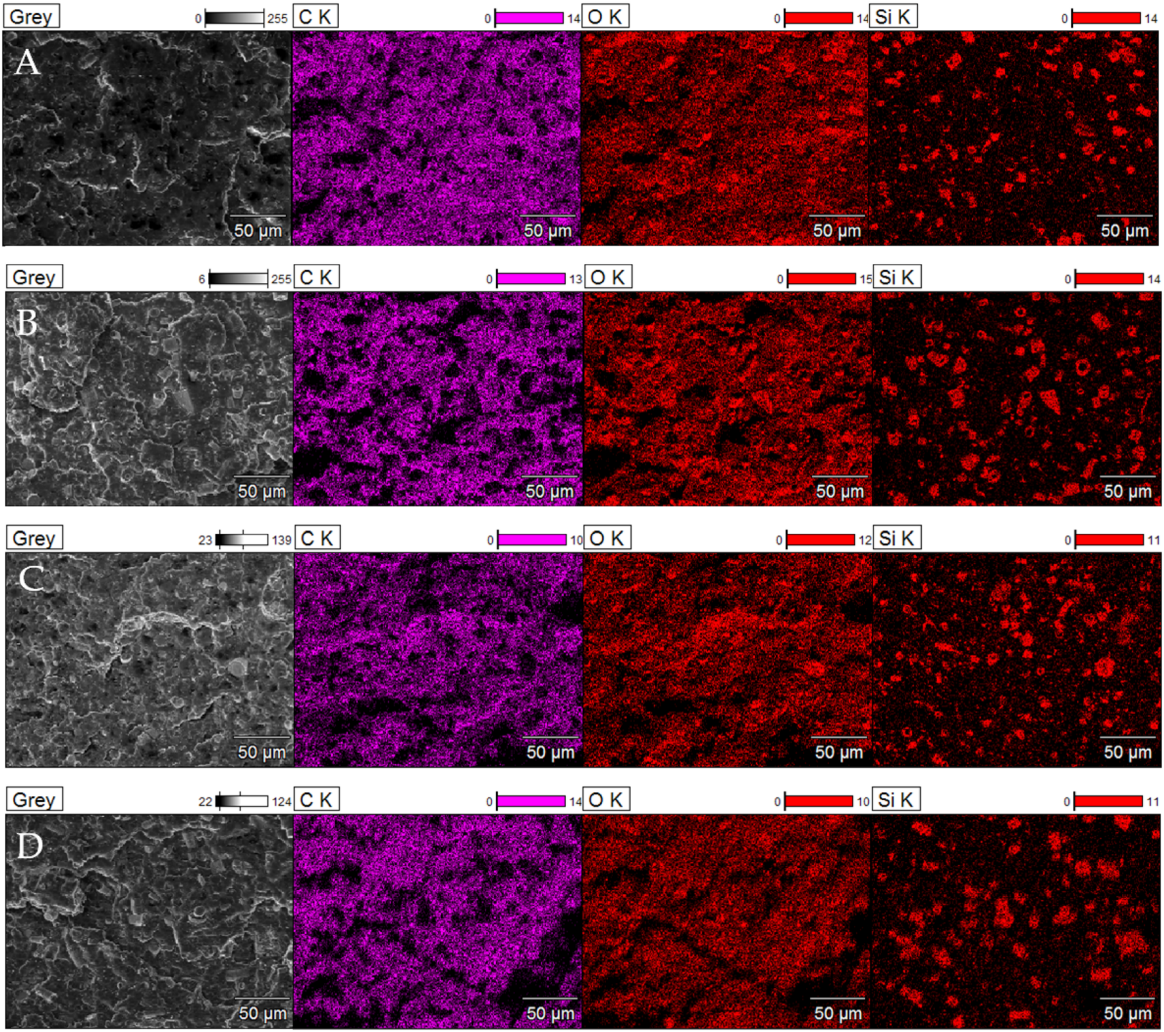
EDS; (**A**) 15B, (**B**) 15B/0.5BW, (**C**) 15B/1BW, (**D**) 15B/0.5SW.

**Figure 15:**
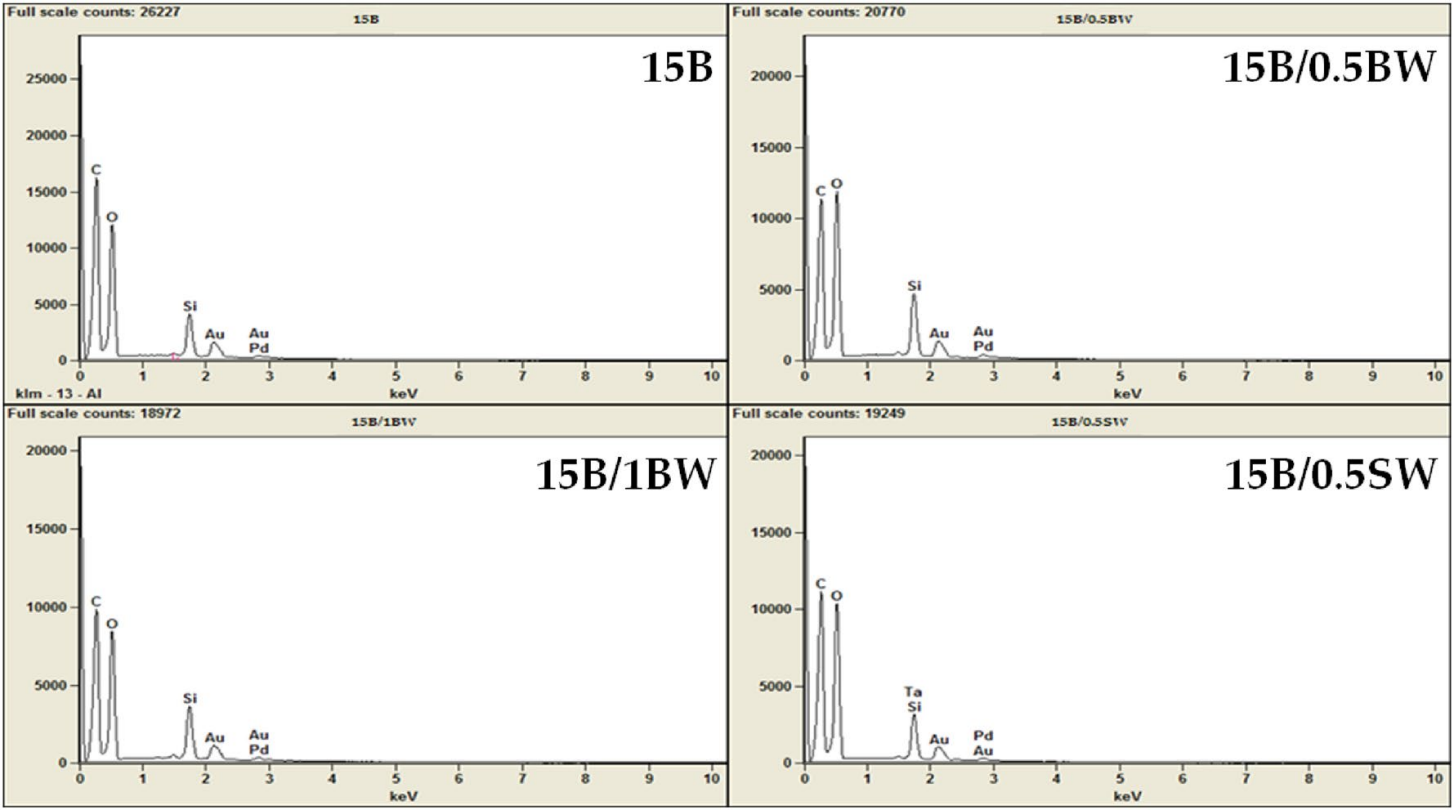
EDS mapping of composite fractures.

Using the SEM/EDS mapping (Figs. 14, 15), the presence of carbon, silicon and oxygen in composites was confirmed. The content of these elements is similar regardless of the modifier used, i.e. the type and concentration of wax. The most important element to observe is silicon, as it allowed to localize diatomite particles embedded in the PLA matrix. On the basis of area on the mapping image, it was possible to detect agglomerates discussed above.

**Figure 16:**
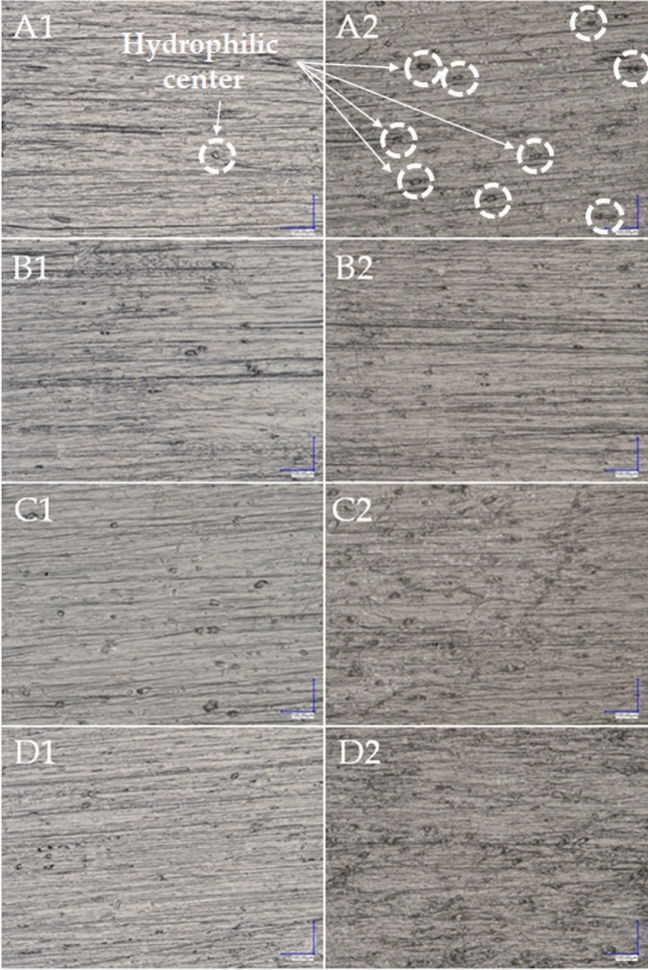
Composite surface; (**A1**) 2.5B, (**A2**) 10B, (**B1**) 2.5B/0.5BW, (**B2**) 10B/0.5BW, (**C1**) 2.5B/1BW, (**C2**) 10B/1BW, (**D1**) 2.5B/0.5SW, (**D2**) 10B/0.5SW.

Using an optical microscope we were able to observe the surface features and composite fractures on a microscale to determine the effects of varying diatomite concentration and the type and quantity of wax added on the microstructural properties of composites. Figure 16 shows the images taken from the surfaces of the samples. Systems with 2.5% and 10% diatomite were studied in detail. The surface of all the composites with the lowest filling is very similar. Numerous furrows are the result of wear on the injection mould. Furthermore, even at low concentrations of diatomite, its small agglomerations are visible, which become larger with increasing concentration (Fig. 16). In systems filled with 10% of diatomaceous earth, numerous agglomerations of DE can be seen (Fig. 16A2), which were more dispersed in the polymer matrix after the addition of 0.5% beeswax (Fig. 16B2). The addition of 1% of beeswax and the addition of synthetic wax do not improve the performance of the surface structure of the composites. In the case of system 10B/1BW, there are also slight ripples on the surface, which may indicate that the excess wax has penetrated outside the composite.

**Figure 17:**
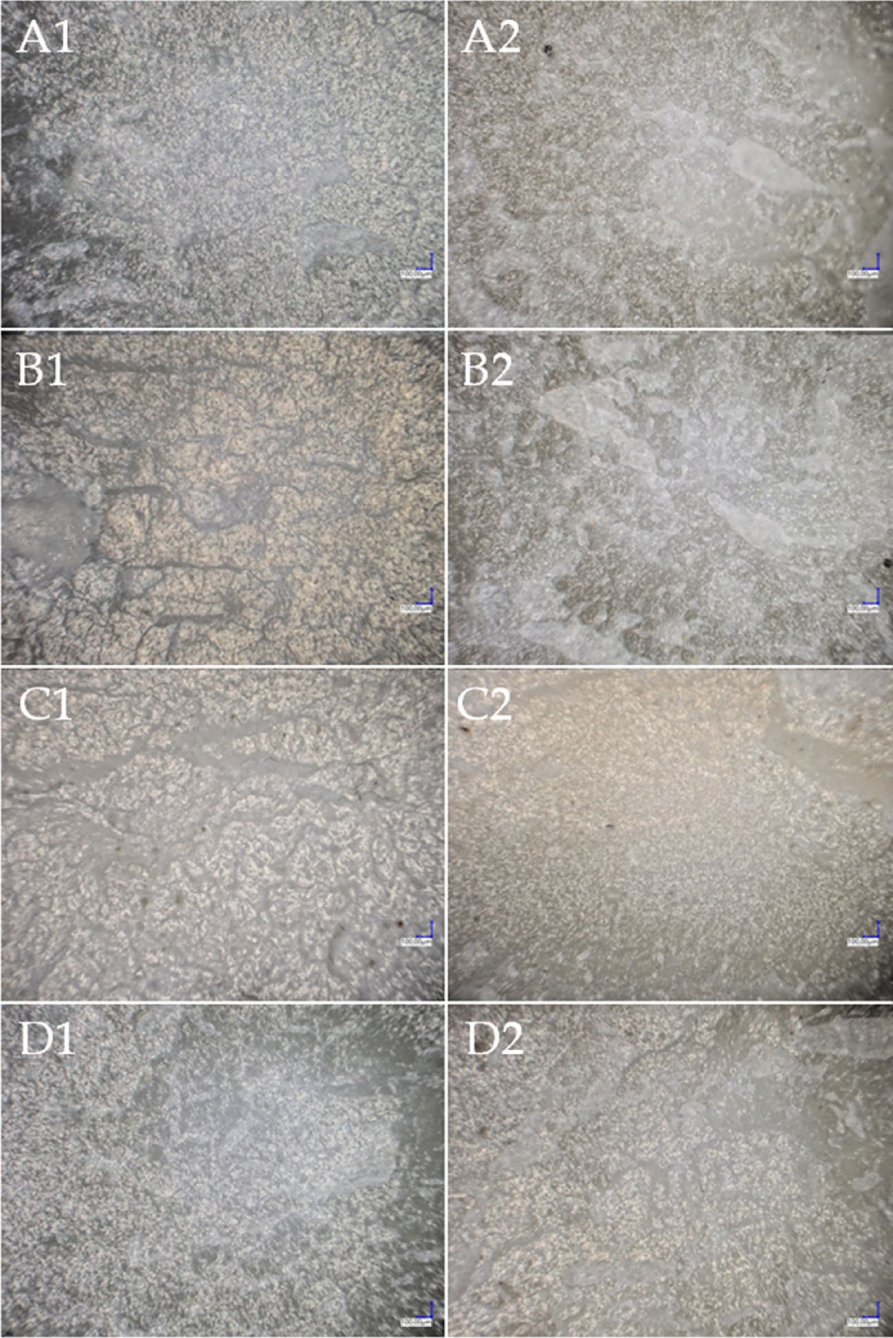
Composite fractures; (**A1**) 2.5B, (**A2**) 10B, (**B1**) 2.5B/0.5BW, (**B2**) 10B/0.5BW, (**C1**) 2.5B/1BW, (**C2**) 10B/1BW, (**D1**) 2.5B/0.5SW, (**D2**) 10B/0.5SW.

Observation of the composite fractures also showed the effect of the modification on the behaviour of the composite surface during the impact strength testing (Fig. 17). In low-fill systems, especially in the system modified by adding 0.5% beeswax, numerous furrows and ripples are present on the material. However, no clear edges caused by the impact are visible. There are also numerous whitened areas, which probably origin from diatomaceous earth, and are due to insufficient dispersion of the filler in the polylactide matrix. This effect is more pronounced for systems containing 10% DE, where, as expected, the filler dispersion is even lower, due to the high addition of diatoms. In contrast, the addition of 1% beeswax seems to compensate for this effect and probably has a positive effect on the dispersion of the filler in the matrix, as shown in Fig. 17C2. The structure of this composite is significantly smoother than that of the others and no whitening or other defects are present.

**Figure 18:**
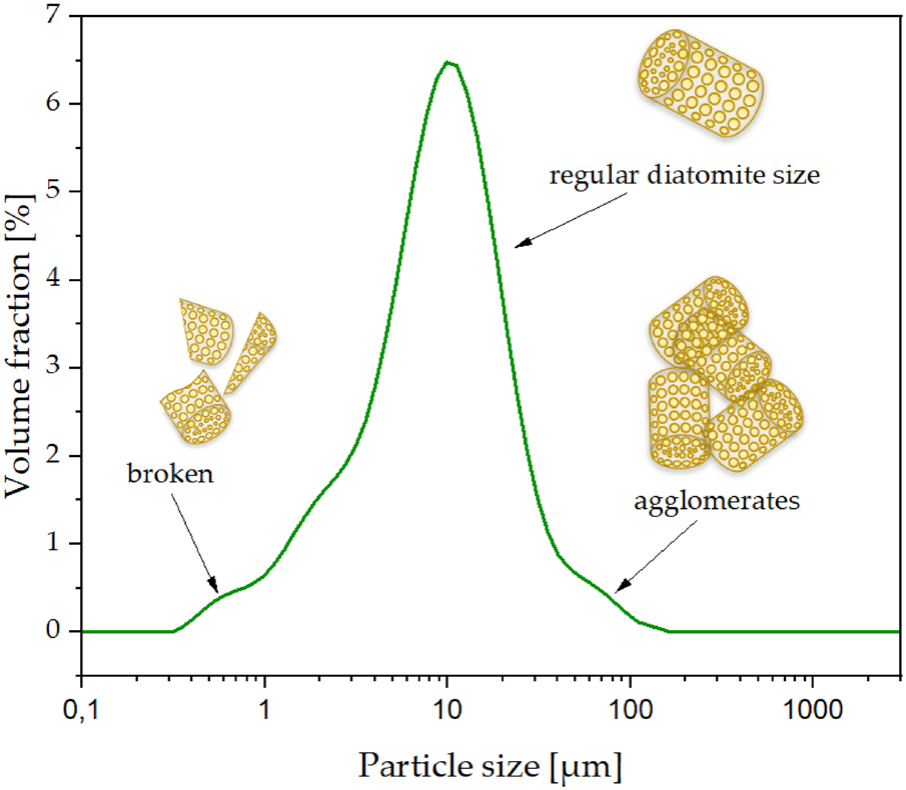
Particle size distribution of the original diatomite.

The use of the light microscope for viewing composites containing the mineral filling is an excellent complement to the SEM, with which it is not possible to determine the colour of the individual components of the composite, and therefore the individual phases of the composite cannot be easily identified. Using SEM, composite components can often be interpreted as a uniform structure, whereas a light microscope shows different phases. In contrast, it is not possible to distinguish small (crushed) particles of diatomite cannot be distinguished from large particles (not crushed) (Fig. 18). Only when all these techniques are combined, we can get a full picture of the diatomite-filled composites. Diatomite takes on different colours depending on the fragmentation (crushed frustules appear whiter).The fact that the diatomite colour changes according to particle size is due to differences in the refraction and the wavelength of the light that the diatomite absorbs. Under the light microscope, the diatomite takes on a white colour, while the polymer (polylactide) takes on a slightly yellowish colour, which is clearly visible in the photos of the composite fractures (Fig. 17), and on this basis it is easy to identify the dispersion of the filler in the polymer matrix.

## Conclusions

The modification of polylactide with diatomaceous earth and the addition of waxes had a positive effect on the processing, microstructural, mechanical and surface properties of the composites. Already an addition of 0.5% beeswax resulted in a significant improvement of the processing properties not only compared to neat PLA, but also to PLA/DE with different filler concentrations. Moreover, the addition of natural wax also contributes to the flexural strength and impact resistance of composite materials. These properties vary depending on the concentration of the additive used, so it is possible to adjust mechanical parameters according to given requirements. The use of the above additives has improved the hydrophilic and hydrophobic properties of the polylactide surface and enabled the production of hydrophobic composites. It was also found that diatomite used in low concentrations results in an increase in hydrophobic properties due to the geometry of the composite surface, while the higher loading of DE results in the formation of hydrophilic centres at the boundary of the composite surface and a decrease in the contact angle. The effects of the waxes on the dispersion of the fillers in the matrix and the changes in the thermal stability of the composites were also noted. The addition of synthetic wax used for comparative purposes also has advantages. Its content has a positive effect on the tensile strength of composites conditioned at room temperature, but natural waxes have an advantage over synthetic waxes as an addition to the composite due to their environmental friendliness. Both the filler and the wax additive have a significant influence on the crystallization of PLA and the type of crystallites formed. Due to application of biopolymer PLA, biofiller diatomite and a fully natural beeswax as a processing aid and functional additive, a material meeting the today’s definition of biocomposite was developed, thus offering a green, environment-friendly option of limited carbon footprint when compared to conventional composites based on petrochemical substrates.

## Data Availability

The datasets used and/or analysed during the current study available from the corresponding author on reasonable request.
